# Independent and combined associations of VOCs exposure and MetS in the *NHANES* 2017–2020

**DOI:** 10.3389/fpubh.2025.1572360

**Published:** 2025-03-21

**Authors:** Xin Gao, Shanshan Xu, Na Lv, Chaokang Li, Ye Lv, Keyi Cheng, Hong Xu

**Affiliations:** Hangzhou Center for Disease Control and Prevention (Hangzhou Health Supervision Institution), Hangzhou, Zhejiang, China

**Keywords:** metabolic syndrome, atmospheric pollution, global health, association, cross-sectional analysis, Volatile organic compounds

## Abstract

**Introduction:**

As a worldwide public health concern, Metabolic syndrome (MetS) seriously endangers human health and life safety. It`s reported that there is a strong association between chemical pollutants and the development of MetS in recent years. Volatile organic compounds (VOCs), the primary emission pollutant in atmospheric pollutants, were closely associated with development of chronic diseases. However, the association between VOCs exposure and MetS is unclear. We aimed to investigate the association between VOCs and MetS and identify the behavioral patterns in which MetS patients may be exposed to VOCs.

**Methods:**

We conducted a cross-sectional data analysis from 15,560 VOC-exposed participants in the *NHANES*. Multivariable logistic regression model, weighted quantile sum (WQS) regression model, and Bayesian kernel machine regression (BKMR) regression model were employed to explore chemical exposure`s independent and combined effects on MetS, respectively.

**Results:**

A total of 2,531 individuals were included in our study, of whom 51.28% had MetS and 48.72% were non-MetS. The logistic regression model identified the association between N-acetyl-S-(N- methylcarbamoyl)-L-cysteine (AMCC), N-acetyl-S-(2-carboxyethyl)-L-cysteine (CEMA), N-acetyl-S-(2- cyanoethyl)-L- cysteine (CYMA) and MetS. In WQS regression analysis, the WQS index was significantly associated with AMCC, trans,trans-Muconic acid (t,t-MA), N-Acetyl-S-(1-cyano-2- hydroxyethyl)- L-cysteine (CYHA), CEMA, 2-Thioxothiazolidine-4-carboxylic acid (TTCA), N-acetyl- S-(3- hydroxypropyl-1-methyl)-L-cysteine (HPMM), CYMA, N-acetyl-S-(3,4-dihydroxybutyl)-L-cysteine (NADB), and N-Acetyl-S-(4-hydroxy-2-methyl-2-buten-1-yl)-L-cysteine (IPM3 cysteine). Finally, the combined association of MetS was positively associated with CEMA and CYMA in the BKMR regression model.

**Discussion:**

In summary, we demonstrated that VOCs and their` metabolism were significantly associated with MetS. Compared results from these three models, CEMA and CYMA were identified as the factors associated with MetS. This study provides a research direction for the mechanism of VOCs that may induce the onset and development of MetS.

## 1 Introduction

Metabolic syndrome (MetS) is classified as a metabolic disorder characterized by central obesity, elevated triglyceride (TG), low high-density lipoprotein cholesterol (HDL-C), hypertension, and impaired glucose homeostasis (1). As one of the most serious global public health problems, MetS induces a series of metabolic disorders and increases the risk of chronic diseases, including cardiovascular disease (CVD), type 2 diabetes (T2D), and other chronic diseases (2, 3). Between the 1980s and 2012, the prevalence of MetS among US adults aged 18 years and older rose by over 35%, and more than one-third of all US adults met the definition and criteria for MetS (4). Meanwhile, the research indicates that MetS and its associated complications pose a relatively great threat to children’s health, with the prevalence of overweight and obesity among children and adolescents on the rise (5). Consequently, it has become essential to investigate the risks that influence the development of MetS to reduce the burden of disease. Previous studies have established that the epidemic of MetS is primarily driven by factors such as physical inactivity, overnutrition, aging, and sleep deficiency. Furthermore, increasing evidence indicates that environmental chemicals, particularly air pollution, may also significantly contribute to the development of chronic metabolic diseases (6).

Volatile organic compounds (VOCs) are the primary constituents of air pollutants, which destroy the atmospheric environment and seriously endanger human health. As the research on VOCs and other air pollutants deepens, it causes a variety of chronic diseases, such as chronic obstructive pulmonary disease, asthma, bronchiolitis, and CVD (7). Specifically, VOCs may increase the risk of CVD by depleting circulating angiogenic cells (8, 9). Furthermore, chloroethanol causes white adipose tissue (WAT) inflammation and investigated lipolysis, which alters lipid metabolism and WAT-mediated hepatic steatosis due to changes in WAT lipolysis (10). Moreover, the content of urinary VOCs in T2D patients was significantly higher than that of the healthy subjects (11). A statistically significant association was identified between breath acetone and blood acetoacetate, as well as between breath acetone and *β*-hydroxybutyrate, indicating the potential involvement of VOCs in blood glucose metabolism (12). The above studies indicate that VOCs may contribute to the onset of various metabolic diseases associated with MetS. Despite the limited studies exploring the connection between VOCs and MetS, the exact relationship remains unclear. Therefore, we aimed to investigate the association between VOCs and MetS.

## 2 Materials and methods

### 2.1 Data sources

This study was conducted by the National Health and Nutrition Examination Survey (*NHANES*), a nationally representative cross-sectional health examination survey in the United States, which consists of five main components: demographics, questionnaires, laboratory, dietary, and examination data. The data combined the partial data in 2019–2020 (pre-pandemic) with the previous cycle (2017–2018), and included both MetS and non-MetS patients over 18 years old. In addition to essential demographic characteristics, the study included 62 VOCs, body mass index (BMI), blood pressure, fasting plasma glucose/triglycerides (FPG/TG), and high-density lipoprotein cholesterol (HDL-C) were included in the study. The subjects` questionnaire collected information on their height, weight, smoking history, alcohol consumption, income level, and marital status. All participants provided complete written informed consent, and the study was approved by the Internal Review Board (National Institute of Environmental Research, Environmental Health Research Division-1805; IRB NIER 1805). The guidelines and regulations of the Declaration of Helsinki conducted all methods of this study. The distribution of other VOCs concentrations in urinary and serum samples is shown in Supplementary Table S1.

### 2.2 Participant selection

**Figure 1:**
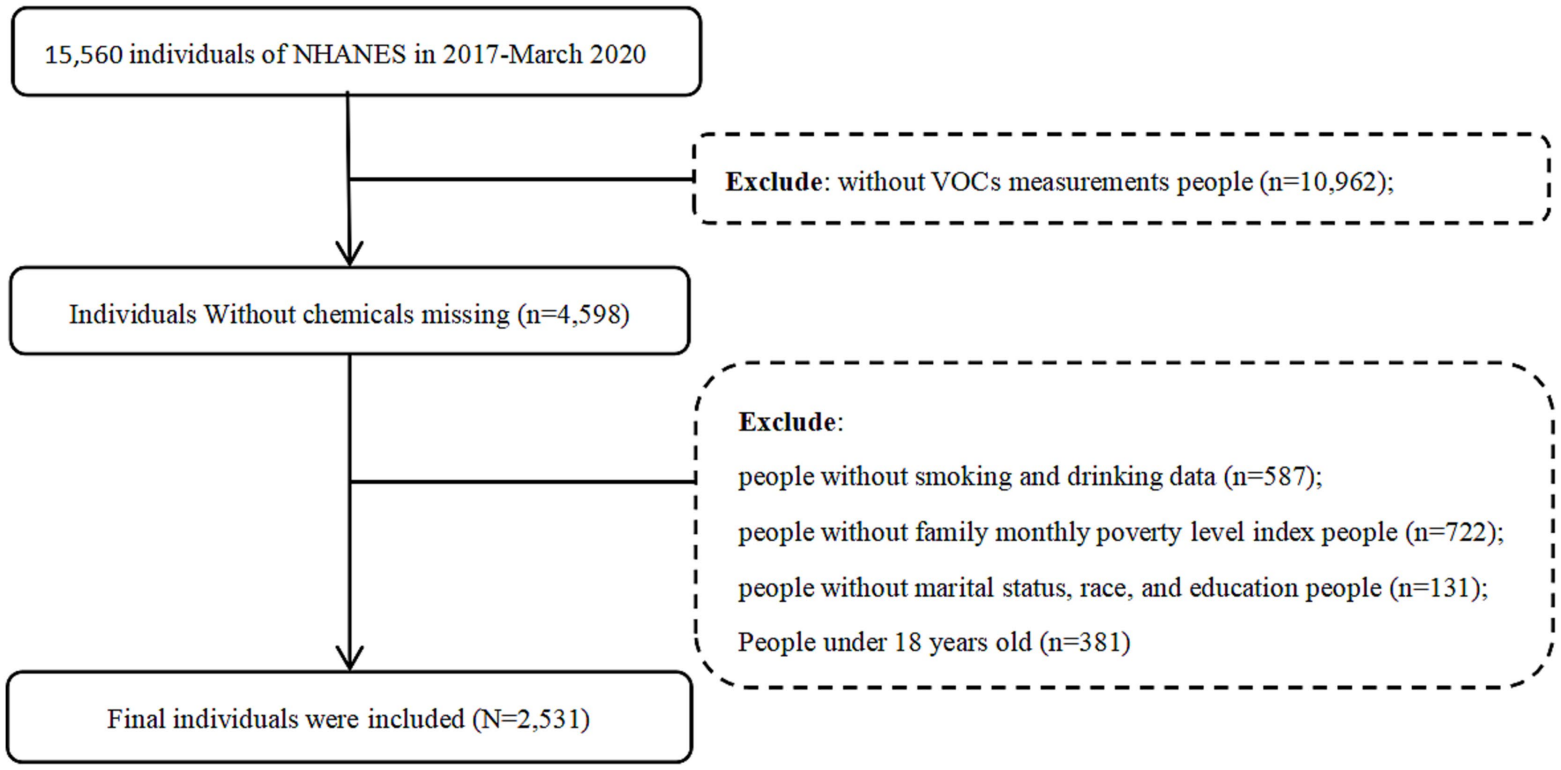
The flow chart for determining the final subjects is included.

We extracted data for a total of 15,560 individuals from *NHANES* (2017-March 2020 pre-pandemic). Following the exclusion criteria, 2,531 participants were eventually included in our study. The flowchart that explains the screening process is shown in Figure 1. Considering data representativeness, we excluded subjects with missing values and those whose lower limit of detection (LLOD) values for urine and serum VOC metabolites in the population exceeded one-fifth of the total population. To evaluate the associations of urine and serum VOCs with MetS, participants with missing data on any of the 62 VOCs (*n* = 10,962) were initially excluded. Participants with missing data on smoking and drinking data (*n* = 587), family monthly poverty level index (*n* = 722), those aged under 18 years (*n* = 381), and core covariates (*n* = 131) were further omitted. A total of 2,531 participants were ultimately included in this study.

### 2.3 MetS definition

We defined MetS based on the *National Cholesterol Education Program/Adult Treatment Panel III* (NCEPATP III) criteria, where an individual was considered to have MetS if they met three or more of the following components (13). As follows: (a) Central obesity (waist circumference > 102 cm for males and > 88 cm for females); (b) Hypertension (blood pressure ≥ 130/85 mmHg or treated with anti-hypertensive drugs); (c) Impaired fasting plasma glucose (fasting glucose ≥5.6 mmol/L or drugs used for treating diabetes); (d) Fasting plasma triglycerides (fasting plasma triglycerides ≥150 mg/dL or treated with drugs for the lipid abnormality); (e) Low HDL cholesterol (males/females <40/50 mg/dL or treated with drugs for this lipid abnormality). Due to the waist circumference data missing, we could not effectively obtain this part of the data. Studies have found a strong association between waist circumference and BMI in patients with MetS (14). An increasing number of studies now utilize BMI as a proxy for central abdominal adiposity rather than waist circumference. Samuel R. Bozeman et al. found that it is possible to construct an alternative estimate of waist circumference using common BMI values in conjunction with demographic factors such as age and sex, particularly when direct waist circumference measurements are unavailable (15). Therefore, we represented centripetal obesity based on BMI, and the subjects were identified obesity when BMI ≥ 30 kg/m^2^ (Supplementary Table S2) (16).

### 2.4 Covariate

Based on the risk factors for MetS in the association analysis study, we sought various risk factors among the behavioral and demographic profiles of participants that may influence the occurrence of Mets. The questionnaires obtained all covariate information, including age (years), gender (male, female), race/ethnicity (Mexican American, other Hispanic, non-Hispanic white, non-Hispanic black, non-Hispanic Asian, other race, multiracial), highest level of education attained (no high school, some high school, high school graduate, some college, college or higher graduate), marital status (married, unmarried, divorced), family poverty level index, smoking (more than 100 cigarettes, less than 100 cigarettes), alcohol consumption (ever had any alcohol, never had any alcohol).

### 2.5 Statistical analysis

All analyses were performed with IBM SPSS Statistics (version 20.0) or R (version 4.2.3). We used the rank sum and Chi-square tests to analyze the subjects’ demographic characteristics, respectively. Through the non-parametric Wilcoxon rank sum test, we identified 62 variables with significant differences as potential risk factors for investigating the association between VOCs and MetS in this study. Since the concentrations of these VOCs data had a skewed distribution, the data were ln-transformed to improve a normal distribution when treated as continuous variables. In addition, the Chi-square test was used to analyze the behavior of MetS patients who may be exposed to VOCs. We adjusted the three statistical models for potential confounding by other known risk factors identified by previous studies, such as age, gender, race, education, family poverty index, smoking, and alcohol consumption. Sampling weights are commonly used to generate representative and unbiased statistics when analyzing survey data. However, further adjustment to the variables used to calculate the sample weights in the regression analysis may reduce the estimate’s precision and even introduce a degree of over-adjustment bias (17). Consequently, the results of this study are presented without sampling weights, similar to those reported in previous studies based on *NHANES* data (18).

#### 2.5.1 Statistical model 1: Generalized linear regression model

First, we fitted the logistic regression model for chemical adjusting for age, gender, and confounding factors. All significant variables in the univariate analysis were considered for the logistic regression analysis based on the backward stepwise method. Considering associations with other exposures, we addressed the possibility that the combined associations of other metabolites of VOCs may result in false-positive or false-negative results by calculating multiple comparisons using Benjamini-Hochberg false discovery rate (FDR) corrections (19). Individual *p*-values (per hypothesis) were ranked from smallest to largest. Adjusted *p*-values were calculated by multiplying the original *p*-value by (m/i), where m is the number of tests, and i is the rank of the specific *p*-value. Then, adjusted *p*-values are compared with the original alpha of 0.05, and the rank of the largest adjusted *p*-value that is less than 0.05 is used to calculate an adjusted alpha level by following the formula 0.05*(i/m) (20). We set the statistical significance to FDR-corrected *p* < 0.05.

#### 2.5.2 Statistical model 2: Weighted quantile sum regression model

Second, the WQS regression model was used to detect the combined effect of the multi-pollutant exposures on MetS, which can solve the problem of high dimensionality and high association between homologous pollutants. We constructed the WQS index of MetS based on quartiles of the metabolites of VOCs and conducted WQS regression analyses by gender, age, education, race, and education with 100 bootstrap samples in each dataset. In this study, bootstrap = 100, quantile = 4, validation = 0.6, seed = 2023. The WQS regression model was used to calculate a weighted linear index (total effect of mixed exposure) representing all VOCs` effects on the MetS. We calculated each VOC weighting index (weight) to represent the specific VOCs` pollutant contribution to the WQS regression model. The fitting model of the WQS regression model was as follows: $g\left(\mu \right)=\beta_{0}+\beta_{1}\left(\overset{c}{\underset{i=1}{\sum }}w_{i}q_{i}\right)+z^{’}\varphi$ (i = 1,2,3...k). Where β was the weight of each component in the environmental mixture, and $\beta_{1}$was the regression coefficient of the weighted quantile sum index (WQS index), which is the overall effect of the environmental mixture. $\beta_{0}$ was the intercept, $z^{`}$ and φrepresented the matrix of covariates and coefficient of covariates. The WQS index was a weighted average across the ensemble step samples using a signal function. We observed the association among multiple exposure factors by drawing a heat map of the association coefficients and used the R package “gWQS” (version 3.0.4) to complete the WQS regression analysis in this study (21).

#### 2.5.3 Statistical model 3: Bayesian kernel machine regression model

Third, we also used the BKMR model to evaluate the combined effect of chemicals on MetS, which can identify non-linear and non-additive relationships within chemicals. The dose–response relationship between single VOCs and MetS was used to determine the risk at different concentrations of VOCs (possible mixing effects). The fitting model of the BKMR regression model was as follows: $Y_{i}=h\left(Z_{i1},\ldots ,Z_{im}\right)+x_{i}\beta +\in_{i}$. Where *h()* was the exposure-response function based on nonlinearity and/or interaction among the mixture components, $Z_{i}$, and *β* represented covariates and their coefficients, respectively. We evaluated the exposure-response relationship between VOCs exposure and potential outcomes by obtaining *h()*. When *h()* > 0, the compound promotes the occurrence of MetS and, conversely, inhibits the occurrence of MetS (22). Moreover, we calculated the posterior inclusion probability (PIP), which is the probability that a spike in the posterior sample and a plate variable selector will include a particular contaminant in the mixture in the model. To determine the importance of each environmental pollutant for the study outcome, a threshold of PIP > 0.5 was used (23).

## 3 Results

### 3.1 The association study between VOCs and MetS

**Figure 2:**
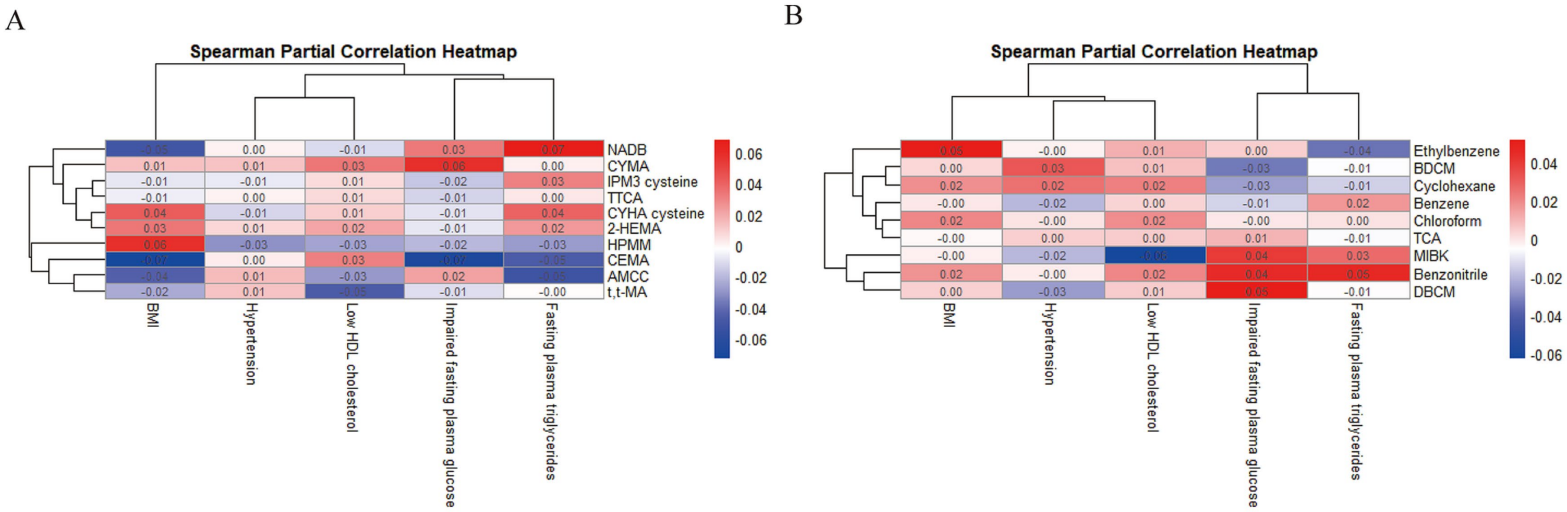
The associations between urinary **(A)** and serum **(B)** VOCs metabolites and components of MetS.

Among the 2,531 subjects, 1,298 were diagnosed with MetS. The demographic characteristics of the subject population are shown in Supplementary Table S3. Overall, age, race, education, smoking, drinking, BMI, systolic blood pressure, diastolic blood pressure, and FPG were significant between MetS and non-MetS participants (*p* < 0.05). As showed that there were differences in BDCM, Benzene, Benzonitrile, Cyclohexane, Chloroform, DBCM, Ethylbenzene, MIBK, 1,1,1-TCA in the serum samples of the subjects. In addition, we also found that there were significant differences in AMCC, CEMA, CYHA cysteine, CYMA, NADB, N-ace-S-(2-hydroxyethyl)-L-cys (2-HEMA), IPM3 cysteine, HPMM, TTCA, and t,t-MA in the urinary samples of subjects (*p* < 0.05). After controlling for other confounding factors, we observed associations between various VOCs in urine and serum and the components of MetS, respectively, in the spearman partial correlation heatmap (Figure 2).

### 3.2 The logistic regression model to assess the association between VOCs and MetS

Next, we included those with significant differences in the rank sum test in the binary logistic regression model to assess the individual effect of each chemical on MetS (Table 1). After adjusting for all the covariates, AMCC and CEMA showed positive associations with MetS, while CYMA was negatively associated among urinary VOCs (*P*_FDR_ < 0.05). Among serum VOCs, Benzene was positively associated with MetS, while Chloroform, DBCM, Ethylbenzene, and Benzonitrile were negatively associated (*P*_FDR_ < 0.05).

**Table 1:** Binary logistic regression analysis of MetS patients exposed to VOCs.

	Influencing factors	*β*	OR	95%CI	*p*-value	*P*_FDR_
Serum VOCs	Benzene	0.186	1.205	1.020–1.423	0.028*	0.0280*
	Chloroform	−0.176	0.838	0.725–0.969	0.017*	0.0204*
	DBCM	−0.482	0.617	0.434–0.879	0.007**	0.0135*
	Ethylbenzene	−0.310	0.733	0.589–0.914	0.006**	0.0135*
	Benzonitrile	−0.524	0.592	0.398–0.879	0.009**	0.0120*
Urinary VOCs	AMCC	0.276	1.318	1.130–1.536	0.000**	0.0000**
	CEMA	0.368	1.445	1.226–1.704	0.000**	0.0000**
	CYMA	−0.165	0.848	0.791–0.908	0.000**	0.0000**
	NADB	−0.211	0.810	0.646–1.014	0.066	0.0660
	2-HEMA	−0.141	0.869	0.748–1.010	0.066	0.0660
	t,t-MA	0.090	1.095	1.006–1.192	0.037*	0.0518

*p-value<0.05, **p-value<0.01.

### 3.3 The WQS regression model to assess the association between VOCs and MetS

**Figure 3:**
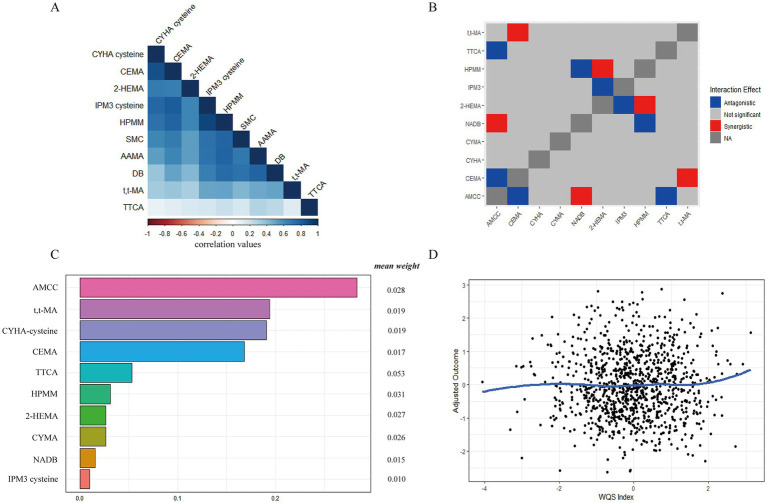
The relative contribution of urinary VOCs to MetS risk based on the WQS regression model. **(A)** The heat maps of urinary VOCs association coefficient; **(B)** The synergistic and antagonistic effects of the interaction of different urinary VOCs by interaction plots; **(C)** The relative contribution of urinary VOCs on MetS. **(D)** The dose–response relationship between urinary VOCs and MetS.

By observing the VOCs association coefficient heat map (Figure 3A), we found strong associations among urinary VOCs. Similarly, we also observed a few associations among serum VOCs (Supplementary Figure S1A). Notably, a distinct overlap was observed in the dark blue region, indicating potential serious collinearity between variables. By drawing interaction plots, we observed distinct synergistic and antagonistic effects among serum or urinary VOCs. In urinary samples, CEMA and t,t-MA, NADB and AMCC, as well as HPMM and 2-HEMA exhibited synergistic effects (Figure 3B). Conversely, antagonistic effects were noted between AMCC and CEMA, TTCA and HPMA with NADB, and 2-HEMA with IPM3. In serum samples, we observed that Benzene and Ethylbenzene with Benzonitrile showed synergistic effects (Supplementary Figure S2B).

Next, we discussed the mixed exposure of VOCs to the influence of the MetS and the relative contribution of various factors (weight) by the WQS regression model. As shown in Figure 3C, after adjustment for the other confounders, the WQS index of mixed urinary VOCs was positively associated with MetS [WQS index = 0.406, *95%CI* (0.219 ~ 0.593), *p* = 0.000]. The weight of urinary VOCs were AMCC (0.283), t,t-MA (0.194), CYHA cysteine (0.191), CEMA (0.168), TTCA (0.053), HPMM (0.031), CYMA (0.026), NADB (0.015), and IPM3 cysteine (0.010), respectively. And the gradual increase in urinary VOCs positively correlated with MetS (Figure 3D). The WQS index of mixed serum VOCs was not significantly associated with MetS [WQS index = −0.528, *95%CI* (−1.137–0.080), *p* = 0.089] in Supplementary Figure S1C. In the dose–response relationship, the gradual increase in serum VOCs negatively correlated with MetS (Supplementary Figure S1D).

### 3.4 The combined and individual effects of exposure to VOCs on MetS by the BKMR model

Except for the possible collinearity of various chemical compounds (combined effect), there were potential non-linear and non-additive relationships within VOCs. Table 2 summarizes the PIPs of VOCs in the BKMR analysis, and we found that CEMA and CYMA contributed the most to the mixed association of VOCs with MetS (PIPs = 1.000). At the same time, AMCC contributed to the higher mixed association of VOCs with MetS (PIPs>0.500).

**Table 2:** PIPs in BKMR model in NHANES 2017–2020.

	Variables	PIPs		Variables	PIPs
Serum VOCs	BDCM	0.000	Urinary VOCs	AMCC	0.620
	Benzene	0.060		CEMA	1.000
	Benzonitrile	0.000		CYHA cysteine	0.336
	Cyclohexane	0.000		CYMA	1.000
	Chloroform	0.110		NADB	0.204
	DBCM	0.202		2-HEMA	0.384
	Ethylbenzene	0.000		IPM3-cysteine	0.152
	MIBK	0.244		HPMM	0.204
	1,1,1-TCA	0.000		TTCA	0.310
	/	/		t,t-MA	0.406

**Figure 4:**
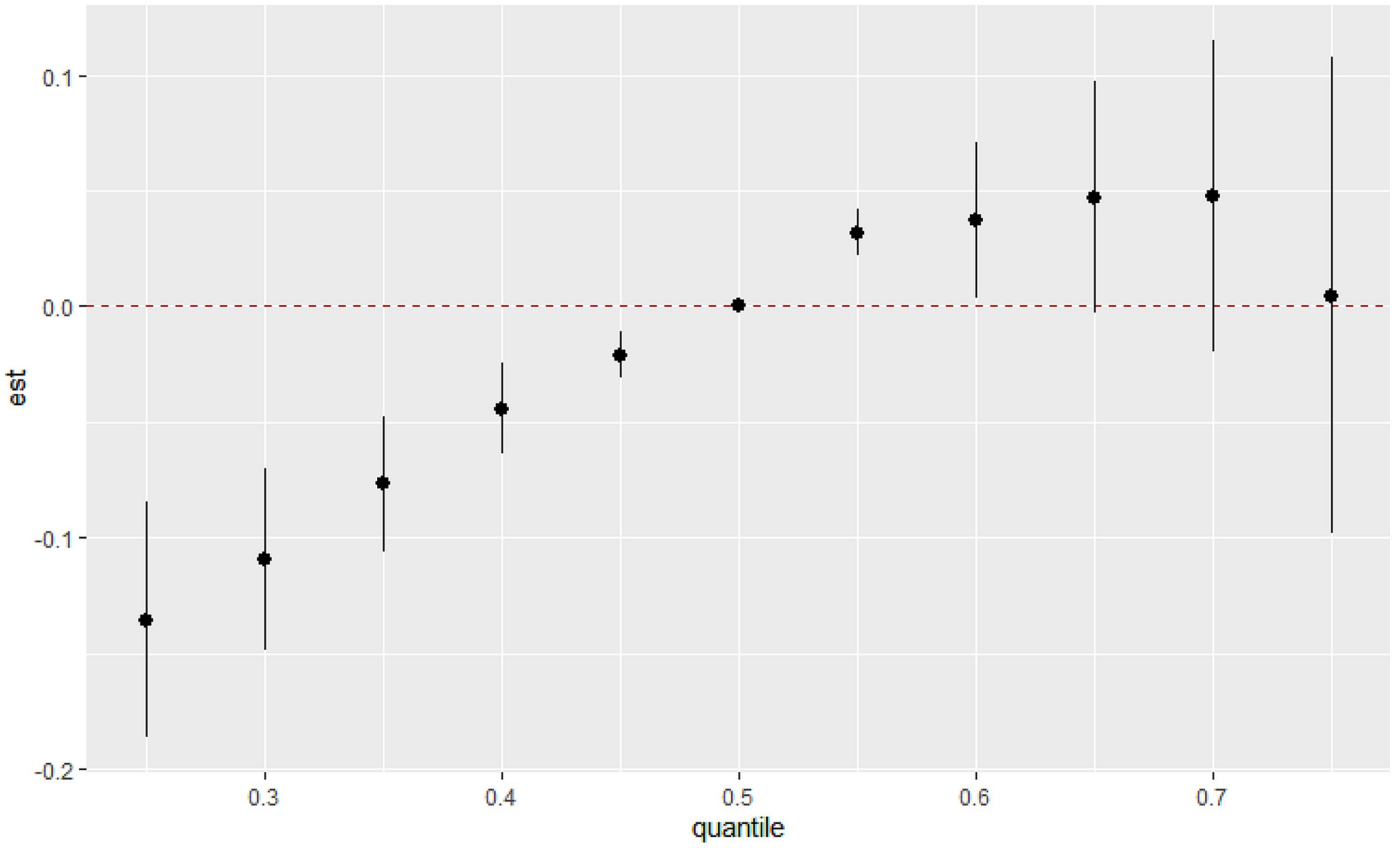
Joint effect (95% CI) of the urinary VOCs mixture on MetS by the BKMR regression model when all the chemicals at particular percentiles were compared to all the chemicals at their 50th percentile.

The overall associations between the chemical mixture of urinary VOCs and the latent continuous outcome are shown in Figure 4. Although confidence intervals were wide, the latent continuous outcome of MetS showed a significant increase when all the chemicals were at their 55th or 60th percentile, compared to their 50th percentile, indicating a significant, positive association with MetS. On the contrary, no statistically significant difference was found in the MetS, and there was an overall decreasing trend in serum VOCs (Supplementary Figure S2).

**Figure 5:**
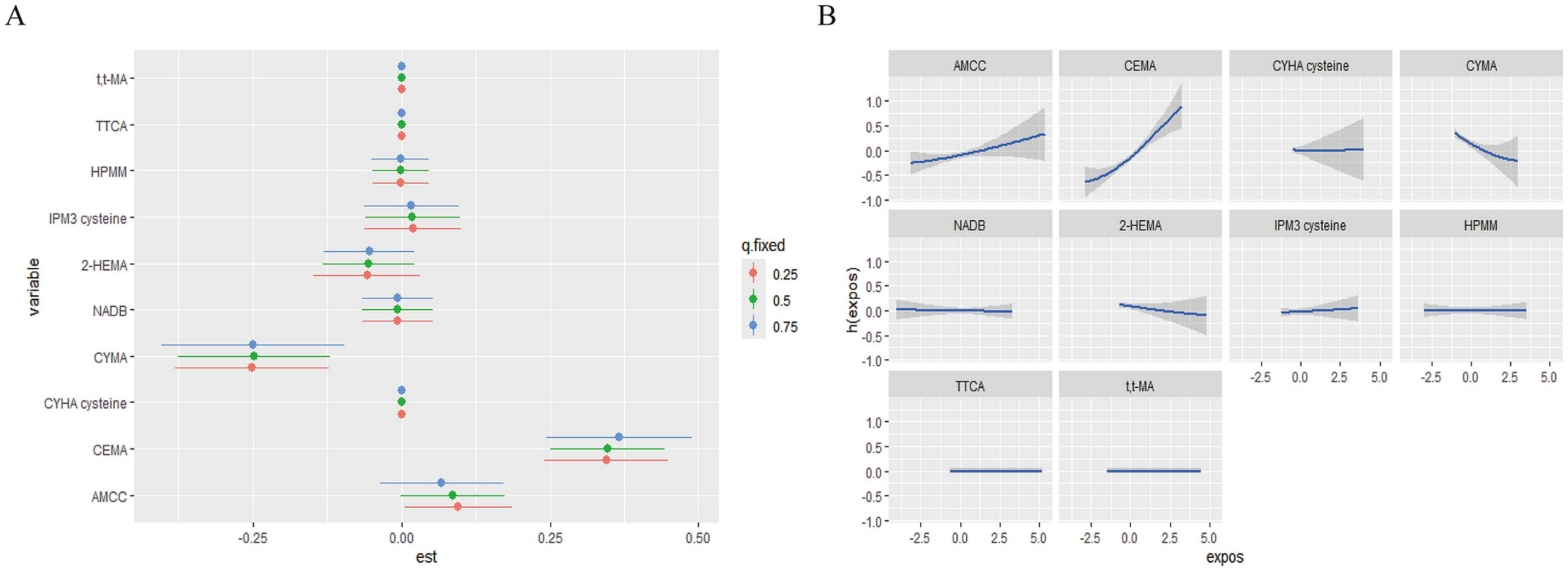
The single pollution model and univariate exposure-response relationship in the BKMR regression model. **(A)** The effect of urinary VOCs on MetS in the single pollution model. **(B)** Univariate exposure-response function (95% CI) between the single urinary VOC concentration and MetS. h(expos) can be interpreted as the relationship between chemicals and MetS. The results were assessed by the BKMR model adjusted for age, gender, race, education, family poverty index, smoking, and alcohol consumption, and ln-transformed creatinine.

Next, we calculated the continuous change in the risk of MetS associated with an interquartile range (IQR) increase in a single pollutant level when the other urinary VOCs are fixed at the 25th, 50th, and 75th percentiles (Figure 5A). A significant association with MetS was observed for CEMA when other elements were set at their 25th, 50th, and 75th percentile, respectively (*p* < 0.05). Specifically, we found that the effects of CEMA on MetS rose as the other VOCs increased from their 25th to 75th percentiles. Additionally, we also observed a negative association between CYMA and MetS when the other urinary VOCs were set at their 25th, 50th, and 75th percentile, respectively (*p <* 0.05). Finally, the remaining serum VOCs were not significantly associated with MetS at the 25th, 50th, or 75th percentile, possibly due to the presence of interactions among VOCs mixtures (highly overlapping confidence intervals, Supplementary Figure S3A).

We estimated both univariate concentration-response (C-R) functions to further investigate the potential non-linear C-R relationship and possible interaction of the VOCs mixture. When the remaining pollutants were kept at the 50th percentile, the promoting effects of AAMC and CEMA on MetS gradually increased, whereas CYMA had an opposing effect (Figure 5B). Meanwhile, the promoting effect of MIBK on MetS gradually increased when the remaining pollutant serum VOCs remained at the 50th percentile (Supplementary Figure S3B). No other discernible patterns (discontinuous trends) were found. Finally, the exposure-response curves of elements suggested the negative trends of associations between CYMA and MetS and positive trends of associations between AAMC, CEMA, and MetS.

### 3.5 The subgroup analysis of urinary VOCs and MetS

In the WQS regression and BKMR regression analyses, given the lack of significant associations between serum-based VOCs and metabolic syndrome (MetS), we focused the subgroup analyses on the relationship between urinary VOCs and MetS. Our findings indicate that in young individuals, Mexican Americans, individuals living in deep poverty, and smokers, exposure to VOCs was more likely to be associated with an increased risk of MetS (Table 3).

**Table 3:** The Subgroup analysis of urinary VOCs and MetS by WQS regression model.

Subgroup	Variables	β(95%CI)	*p*
Gender	Male	0.255(0.018, 0.492)	0.035*
	Female	0.442(0.161, 0.723)	0.002*
Age group	18–44 years old	0.378(0.094, 0.662)	0.009**
	45–60 years old	0.292(−0.039, 0.624)	0.084
	Over 60 years old	0.109(−0.211, 0.428)	0.506
Race	Mexican American	0.459(0.042,0.876)	0.031 *
	Hispanic	0.339(−0.552, 1.230)	0.456
	Non-Hispanic White	0.354(−0.015, 0.723)	0.060
	Non-Hispanic Black	−0.100(−0.482, 0.281)	0.606
	Non-Hispanic Asian	–	–
	Others race	0.313(−0.418, 1.044)	0.401
	Mexican American	0.117 (−1.024, 1.257)	0.841
The monthly poverty level of a family	Mild poverty	−0.069(−0.667, 0.529)	0.821
	Moderate poverty	0.048(−0.347, 0.443)	0.811
	Deep poverty	0.454(0.194, 0.713)	0.000**
Smoking	No smoking	0.131(−0.114, 0.376)	0.293
	Smoking	0.441(0.101, 0.781)	0.011*

*p-value<0.05, **p-value<0.01.

### 3.6 The subjects’ characteristics of VOCs-exposed

Moreover, we analyzed the subjects’ behavior to investigate the source of VOCs exposure in MetS patients in Supplementary Table S4. There were significant differences in the behavior patterns between MetS and non-MetS subjects when using moth balls or toilet deodorant, inhaling smoke the last time, and spending time in the pool hot tub tidy bathroom.

### 3.7 Sensitivity analysis

The linear regression and WQS regression [WQS index = 0.416 *95%CI* (0.221–0.610), *p* = 0.000] were rerun in this study after removing outliers (Supplementary Table S5). Overall, the results of the sensitivity analysis were robust.

## 4 Discussion

Our study combined three regression models to estimate the combined effects of a mixture to evaluate the association of VOCs comprehensively and MetS through participants` serum and urinary samples, respectively. Firstly, the logistic regression model identified the association between AMCC, CEMA, CYMA, and MetS. Secondly, the WQS regression model identified the roles of AMCC, t,t-MA, CYHA cysteine, CEMA, TTCA, HPMM, CYMA, NADB, and IPM3 cysteine in the occurrence and development of MetS. Finally, the combined association of MetS was positively associated with CEMA and CYMA in the BKMR regression model. Compared to the results from these three models, CEMA and CYMA were identified as the important factors associated with MetS.

Acrolein was a chemical used as an intermediate reactive aldehyde in the chemical industry, where it was used to synthesize many organic substances (24). Meanwhile, it was found in emissions from the combustion of fuels, wood, and plastics, ambient air pollution, and electronic cigarette vapor (25). Furthermore, as a byproduct of endogenous lipid peroxidation, acrolein’s harmful effects were mediated through various mechanisms, including DNA damage, ROS formation, protein adduction, endoplasmic reticulum stress, and mitochondrial dysfunction (26). Recent studies have revealed that CEMA, as an acrolein exposure biomarker, could decrease insulin sensitivity and raise fasting insulin FPI, FPG, HOMA-insulin resistance, risks of prevalent IR, impaired fasting glucose, impaired fasting glucose (IFG), and the risk of type 2 diabetes, respectively (27). It revealed that acrolein exposure may impair glucose homeostasis and increase T2D risk via mediating mechanisms of heme oxygenase-1 activation, lipid peroxidation, protein carbonylation, and oxidative DNA damage. Hongying Dai et al. reported that the urinary CEMA concentration in non-smokers and smokers without respiratory diseases was 99 ng/mL and 197.1 ng/mL, respectively (28). Despite spatial variations in exposure levels across different regions, we observed that the urinary CEMA among subjects was similar to those reported in the studies above, and the urinary CEMA concentration in MetS patients (114 ng/mL) was significantly elevated compared with that of the control group (91.9 ng/mL). After adjusting for high-risk factors such as smoking and drinking, we identified a substantial positive association between CEMA and MetS, indicating that CEMA may contribute to the development of MetS by promoting oxidative DNA damage, escalating lipid peroxidation, and triggering abnormal glucose metabolism. Acrylonitrile (ACN) is a colorless volatile liquid mostly present in tobacco smoke, and its exposure has been demonstrated to increase oxidative stress in animal studies. In a study of the association between CYMA, oxidative stress product 8-OHdG, and CVD risk factors, the urinary CYMA concentration was 4.67 μg/L in a cohort of adolescents and young adults in Taiwan. Although CYMA was not significantly associated with CVD, CYMA, as the crucial metabolic byproduct of acrylonitrile was correlated with increased levels of 8-OHdG at higher levels (29). A kinetic study of ACN uptake and CYMA excretion found that the average respiratory retention of ACN was 52, and 21.8% of the retained ACN was excreted as CYMA in urine (30). Elimination approximated first-order kinetics with a half-life of about 8 h. Thus, it cannot be used as an individual index of exposure. Despite recent studies that have explored ACN exposure through multiple linear regression models to fit CYMA concentrations with other confounding factors as predictor variables, the relationship between CYMA and ACN exposure remains inadequately demonstrated (31). The CYMA exposure level of the subjects reported here (1.465 ng/mL) was lower than those typically reported by the rest of the biomonitoring studies. We observed a significant negative association between CYMA and MetS, suggesting a potential association between these ACN metabolites and the occurrence and development of MetS. However, further research is needed to establish a causal relationship.

The exposure pathways of VOCs are closely related to the diverse behavioral patterns of subjects. Emissions originate from a wide range of indoor and outdoor sources, including combustion and evaporation, such as smoking, emissions related to organic solvents, decoration, and household products (32). The emission of VOCs in homes depended on the strength of emission sources, ventilation rates, and the indoor oxidative environment, which reflected differences in chemical use, building materials, and occupant behavior (33, 34). Based on the analysis of the behavior pattern in the VOCs exposed, we deem that banning it is particularly important to prevent MetS by prohibiting indoor smoking, reducing the time spent in the bathroom, paying attention to the emissions of organic solvents, and indoor ventilation. Potential policy implications and public health implications of recommendations on reducing VOCs exposure to prevent MetS. In terms of economic costs and benefits, technology upgrades to accurately quantify and control volatile chemical (VCP) emissions and strengthening industrial abatement measures can help reduce indoor and outdoor air pollution (35). The control of VOCs pollution has a positive significance in reducing the medical costs of chronic diseases. Therefore, industrial upgrading and control of VOC pollution can reduce the disease burden caused by VOC-induced chronic diseases, which has long-term benefits. In terms of health equity, we found that among young people, Mexican Americans, those living in extreme poverty, and smokers, the possibility of VOC exposure being associated with an increased risk of MetS is relatively high in the subgroup analysis (Table 3). This may be since these groups are often exposed to higher concentrations of VOC, such as using inferior decoration materials and having bad living habits, etc. Policies should prioritize protecting vulnerable groups and strengthening health education for these groups.

There are few studies on VOCs and MetS, and the relationship remains unclear. Compared with the study of Dong et al. (15 urinary VOCs), we simultaneously analyzed 62 VOCs among the serum and urinary samples of the subjects to explore the associations between more types of VOCs and different metabolic stages with MetS (36). Based on Supplementary Figure S4, we found that the vast majority of VOCs were highly positively correlated with fasting plasma triglycerides, Low HDL cholesterol, fasting plasma glucose, and Hypertension. Next, we adopted three statistical models to explore the association between VOCs mixed exposure and MetS. Compared to using a single model to explore the impact of co-exposure of compounds on the outcome, we believe that using more models simultaneously can better leverage the advantages of different statistical methods and complement each other’s shortcomings. Somewhat different from the study by Dong et al., we found that CYMA was also identified as a factor associated with MetS by comparison of the results of these three models, except for CEMA.

Meanwhile, we also note the shortcomings and limitations of this research. Although this study explored the association between VOCs and MetS by using three regression models, statistical methods remain insufficient. Firstly, the logistic regression model was widely used to probe into the effects of chemical pollutants on the human body, which cannot solve the mixing and non-collinear interaction between chemical pollutants (37). The WQS and BKMR regression models can better solve this problem and solve non-linear effects and interactions. While the WQS regression model cannot exist simultaneously applied to evaluate the combined effects of chemicals with diverse effect directions, the BKMR regression model may be difficult to understand intuitively and needs more detailed quantitative explanations due to its non-linear and interacting nature (38). Therefore, we can find the contribution of variables through the logistic and WQS regression models and probe into their direction through the BKMR regression model. We compared the results of three regression models to make up for shortcomings in statistical methods selection. Secondly, the confounders, such as smoking and alcohol consumption were controlled in this study, while there are still potential confounders in reality. For example, physical activity was an important protective factor for the prevention and treatment of MetS, and regular physical activity can significantly reduce the risk of type 2 diabetes and CVD (39). As we all know, dietary habits are closely related to the pathogenesis of chronic metabolic diseases. In a cohort study, subjects with poor dietary habits had 1.18 times greater odds for MetS than those adhering to a healthy diet (40). The data on physical activity and diet was not included in this study due to the data missing. Thirdly, sampling weights were used to generate representative and unbiased statistics when analyzing survey data in this study. However, further adjustment of the variables used to calculate the sample weights in this study’s regression analysis may reduce the precision of the estimate and even introduce a degree of over-adjustment bias. Therefore, this study’s results are presented without sampling weights, similar to those reported in previous studies based on *NHANES* (41).

This study utilized a cross-sectional survey of VOC-exposed populations in the *NHANES* database from 2017 to 2019 to investigate the potential association between VOCs exposure and MetS. Although we used three models to explore the association between VOCs and MetS in this cross-sectional study, we only show an association, without being able to confirm causality. Therefore, our additional studies will confirm causality and determine the mechanism of action by experimental research. As research on the pathogenesis of MetS deepens, mitochondrial damage, autophagy, ferroptosis, and other pathways play an important role in the occurrence and development of chronic diseases caused by oxidative stress and lipid peroxidation (42–44). It has been further confirmed in clinical data of MetS patients and the experimental models *in vitro* and *in vivo* that promoting bioenergy and mitochondrial function is the crucial way to prevent the occurrence and development of MetS (45). Excessive consumption of sugars and long-chain saturated fatty acids was closely associated with lipotoxicity and MetS, including Toll-like receptor 4 (Toll4) activation, regulation of peroxisome proliferator-activated receptor *γ* (PPARγ), sphingolipid remodeling, and activation of protein kinase C. These pathways are pivotal in promoting mitochondrial dysfunction, disrupting fatty acid and protein metabolism, and inducing insulin resistance (46). Next, we will focus on the mechanism of Toll4, PPARγ, and protein kinase C in VOCs that induce MetS through mitochondrial damage and autophagy.

## 5 Conclusion

In summary, we demonstrated that VOCs and their` metabolism were significantly associated with MetS. Compared results from these three models, CEMA and CYMA were identified as the factors associated with MetS. This study provides a research direction for the mechanism of VOCs that may induce the onset and development of MetS.

## Data Availability

The original contributions presented in the study are included in the article/Supplementary material, further inquiries can be directed to the corresponding author.
