# Supplementary material for: Independent and combined associations of VOCs exposure and MetS in the NHANES 2017–2020
Source: Front Public Health. 2025 Mar 21;13:1572360. doi: 10.3389/fpubh.2025.1572360 (PMC11968655; doi:10.3389/fpubh.2025.1572360)
Supplement: Supplementary file 1 [file Data_Sheet_1.docx]

**Supplementary Information**

**
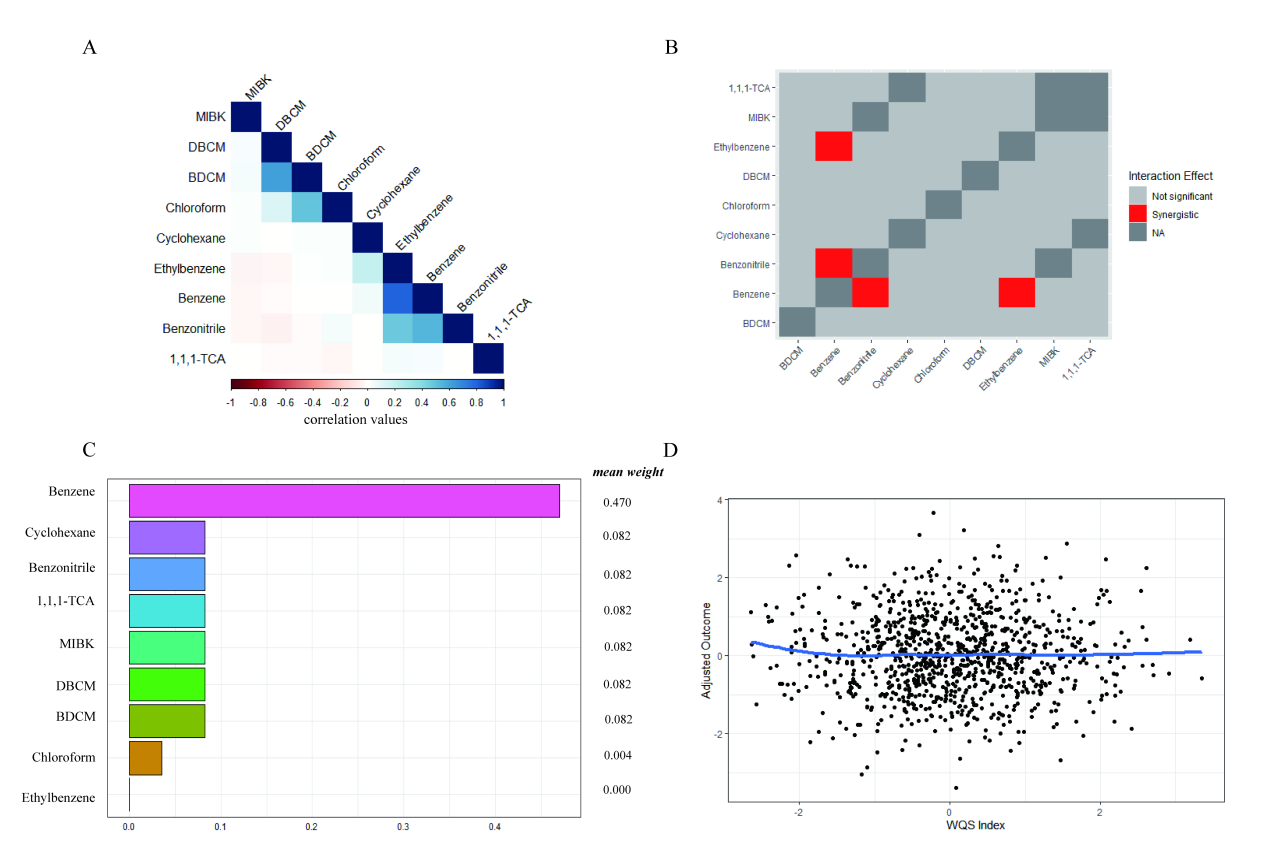
**

**Supplementary Figure S1.** The relative contribution of serum VOCs to MetS risk based on the WQS regression model. (**A**) The relative contribution of serum VOCs to MetS risk based on the WQS regression model. (A) The heat maps of serum VOCs correlation coefficient; (B) The synergistic and antagonistic effects of the interaction of different serum VOCs by interaction plots; (C) The relative contribution of serum VOCs on MetS. (D) The dose-response relationship between serum VOCs and MetS.


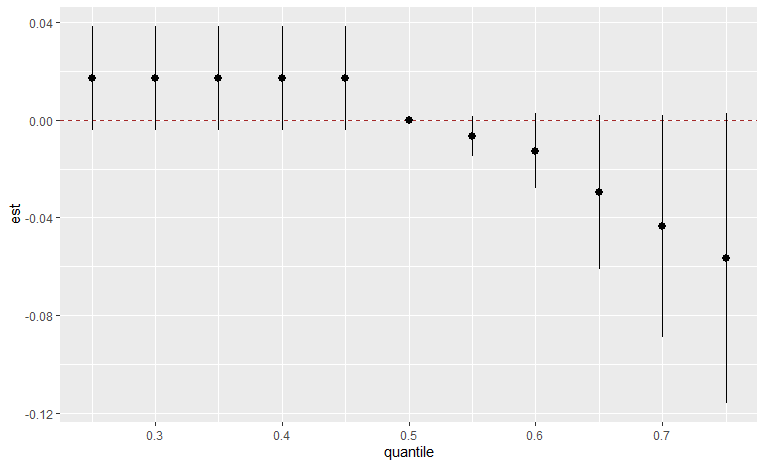


**Supplementary Figure S2.** Joint effect (95% CI) of the serum VOCs mixture on MetS by BKMR model when all the chemicals at particular percentiles were compared to all the chemicals at their 50th percentile.


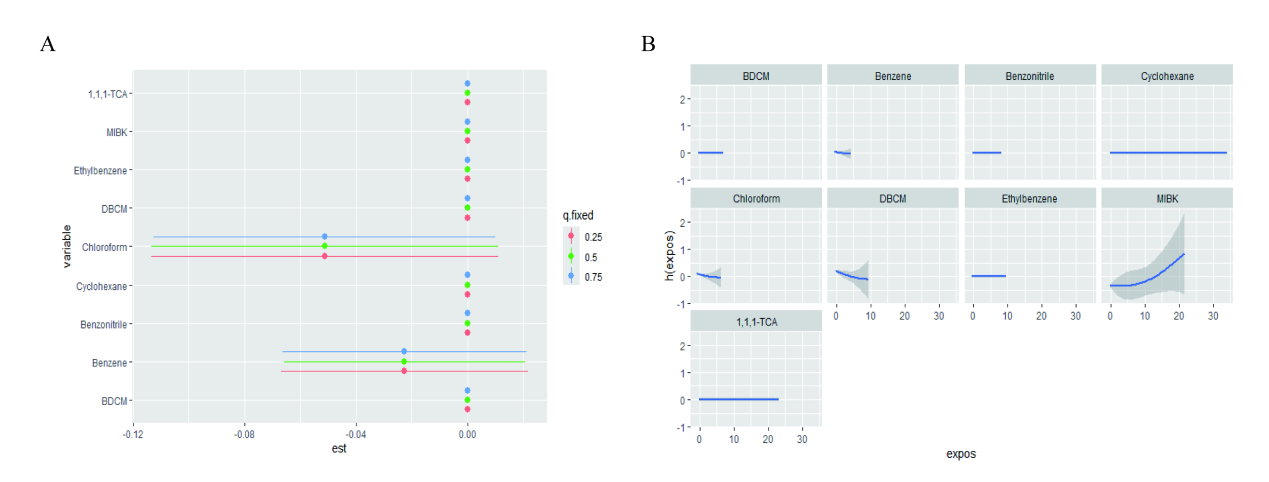


**Supplementary Figure S3.** The single pollution model and univariate exposure-response relationship in the BKMR regression model. (**A**) The effect of serum VOCs on MetS in the single pollution model. (B) Univariate exposure–response function (95% CI) between a single serum VOC concentration and MetS. h(expos) can be interpreted as the relationship between chemicals and MetS. The results were assessed by the BKMR model adjusted for age, gender, race, education, family poverty index, smoking, and alcohol consumption, and ln-transformed creatinine.


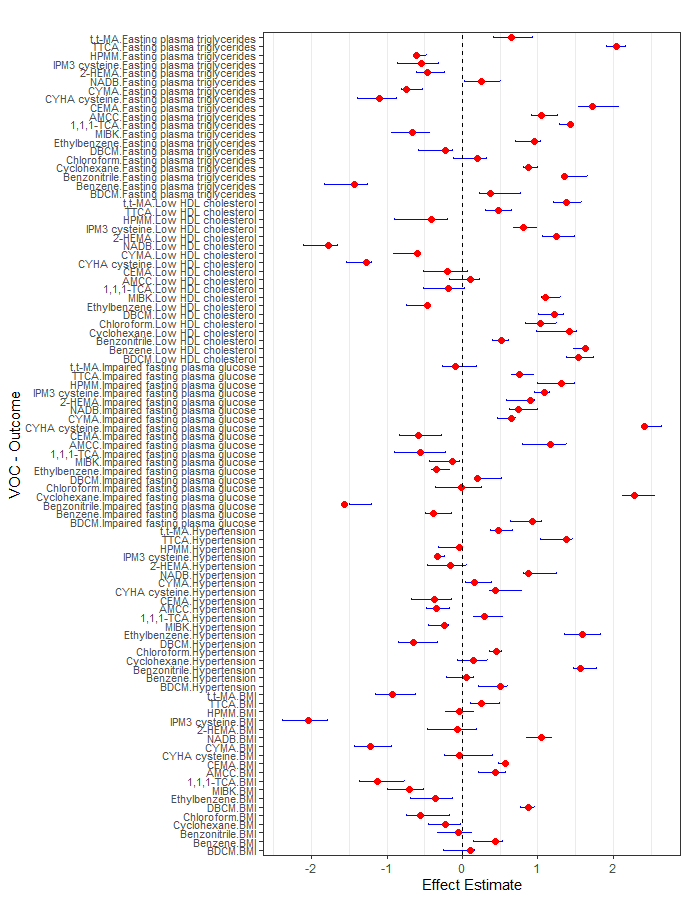


**Supplementary Figure S4.** Association between blood and urine sample species VOC and MetS component**s.**

**Table**

**Supplementary Table S1** Association between single serum or urinary chemical/metabolite concentration and MetS

| **Categories** | **Variables** | **non-MetS (ng/mL)** | **MetS (ng/mL)** | ***P-value*** |
| --- | --- | --- | --- | --- |
| Serum VOCs | DMF | 0.008(0.008,0.008) | 0.008(0.008,0.008) | 0.253 |
|  | 1,1,1,2-Teca | 0.028(0.028,0.028) | 0.028(0.028,0.028) | 1.000 |
|  | Hexane | 0.086(0.086,0.086) | 0.086(0.086,0.086) | 0.655 |
|  | Heptane | 0.071(0.071,0.071) | 0.071(0.071,0.071) | 0.137 |
|  | Octane | 0.071(0.071,0.071) | 0.071(0.071,0.071) | 0.904 |
|  | 1,2-DCB | 0.018(0.018,0.018) | 0.018(0.018,0.018) | 0.330 |
|  | 1,2-DCE | 0.070(0.070,0.070) | 0.070(0.070,0.070) | 0.133 |
|  | 1,3-DCB | 0.018(0.018,(0.018) | 0.018(0.018,(0.018) | 1.000 |
|  | TETRA | 0.034(0.034,0.034) | 0.034(0.034,0.034) | 0.357 |
|  | Bromoform | 0.006(0.006,0.006) | 0.006(0.006,0.006) | 0.097 |
|  | BDCM | 0.004(0.004,0.004) | 0.004(0.004,0.004) | 0.010* |
|  | Benzene | 0.017(0.017,0.052) | 0.017(0.017,0.041) | 0.007* |
|  | Benzonitrile | 0.106(0.106,0.106) | 0.106(0.106,0.106) | 0.000** |
|  | Cyclohexane | 0.014(0.014,0.014) | 0.014(0.014,0.014) | 0.029* |
|  | Chlorobenzene | 0.008(0.008,0.008) | 0.008(0.008,0.008) | 0.533 |
|  | Chloroform | 0.009(0.006,0.015) | 0.008(0.006,0.014) | 0.004** |
|  | DBCM | 0.004(0.004,0.004) | 0.004(0.004,0.004) | 0.016* |
|  | CarbonTetrachloride | 0.004(0.004,0.004) | 0.004(0.004,0.004) | 0.959 |
|  | 1,4-DCB | 0.028(0.028,0.112) | 0.028(0.028,0.106) | 0.968 |
|  | 1,2-Dibromoethane | 0.011(0.011,0.011) | 0.011(0.011,0.011) | 1.000 |
|  | DiethyEther | 0.028(0.028,0.028) | 0.028(0.028,0.028) | 0.330 |
|  | EheylAcetate | 0.112(0.112,0.112) | 0.112(0.112,0.112) | 0.210 |
|  | Ethylbenzene | 0.017(0.017,0.036) | 0.017(0.017,0.029) | 0.018* |
|  | Chloroethane | 0.032(0.032,0.032) | 0.032(0.032,0.032) | 0.534 |
|  | Furan | 0.018(0.018,0.018) | 0.018(0.018,0.018) | 0.574 |
|  | Isobutyronitrile1 | 0.028(0.028,0.028) | 0.028(0.028,0.028) | 0.641 |
|  | Isobutyronitrile2 | 0.028(0.028,0.028) | 0.028(0.028,0.028) | 0.050 |
|  | MethyleneChloride | 0.177(0.177,0.177) | 0.177(0.177,0.177) | 0.654 |
|  | MTBE | 0.007(0.007,0.007) | 0.007(0.007,0.007) | 0.203 |
|  | MIBK | 0.071(0.071,0.071) | 0.071(0.071,0.071) | 0.004** |
|  | Nitrobenzene | 0.226(0.226,0.226) | 0.226(0.226,0.226) | 1.000 |
|  | O-Xylene | 0.017(0.017,0.028) | 0.017(0.017,0.027) | 0.394 |
|  | Trichloroehene | 0.008(0.008,0.008) | 0.008(0.008,0.008) | 0.937 |
|  | 1,1,1-TCA | 0.007(0.007,0.007) | 0.007(0.007,0.007) | 0.024* |
|  | 1,1,1,-Trifluorotoluene | 0.028(0.028,0.028) | 0.028(0.028,0.028) | 1.000 |
|  | Tetrahydrofuran | 0.088(0.088,0.088) | 0.088(0.088,0.088) | 0.940 |
|  | 1,2,3-TCP | 0.028(0.028,0.028) | 0.028(0.028,0.028) | 1.000 |
|  | VinylBromide | 0.032(0.032,0.032) | 0.032(0.032,0.032) | 1.000 |
|  | BTEX | 0.047(0.024,0.110) | 0.045(0.024,0.092) | 0.078 |
| Urinary VOCs | 2-MHA | 22.80(10.20,62.25) | 23.50(10.87,61.45) | 0.474 |
|  | 3/4-methipurc acd | 108.0(52.55,310.00) | 120.0(60.20,264.25) | 0.092 |
|  | AAMA | 58.9(28.65,123.00) | 54.15(29.10,105.00) | 0.087 |
|  | AMCC | 122.0(58.2,249.50) | 150.50(76.25,299.00) | 0.000** |
|  | 2A4CA | 109.0(49.80,221.50) | 107.0(49.30,222.25) | 0.883 |
|  | SBMA | 6.02(3.350,11.900) | 6.66(3.36,13.10) | 0.125 |
|  | AcPrCys | 4.56(1.44,13.15) | 3.85(1.438,11.725) | 0.141 |
|  | CEMA | 91.90(46.60,174.00) | 114.00(55.875,216.00) | 0.000** |
|  | CYHA | 1.84(1.84,1.84) | 1.84(1.84,1.84) | 0.038* |
|  | CYMA | 1.7(0.702,25.05) | 1.465(0.655,5.738) | 0.006** |
|  | NADB (DHBMA) | 345.00(191.00,546.00) | 379.00(231.00,576.00) | 0.000** |
|  | 2C2H | 6.65(6.65,15.30) | 6.65(6.65,14.30) | 0.740 |
|  | 2-HEMA | 0.559(0.559,1.66) | 0.559(0.559,1.25) | 0.000** |
|  | 2-HPMA | 31.20(16.20,64.50) | 32.80(16.80,65.33) | 0.235 |
|  | 3-HPMA | 266.0(137.50,570.0) | 258.0(137.00,545.0) | 0.884 |
|  | IPM3 cysteine | 3.79(1.64,10.00) | 4.25(1.98,9.615) | 0.031* |
|  | Mandelicacid | 144.00(76.90,252.50) | 147.0(82.0,238.25) | 0.597 |
|  | NAS3-hydrxy-2-butenyiLcys | 4.45(2.32,10.45) | 4.67(2.53,10.03) | 0.278 |
|  | PGA | 229.0(127.00,374.50) | 235.0(139.0,379.25) | 0.176 |
|  | HPMM | 213.00(118.00,432.00) | 240.00(140.00,443.00) | 0.007** |
|  | TTCA | 7.90(7.90,18.50) | 7.90(7.90,22.8) | 0.001** |
|  | t,t-MA | 46.4(16.45,113) | 48.55(21.675,114) | 0.027* |
|  | PMA | 0.106(0.106,0.298) | 0.106(0.106,0.303) | 0.414 |

Abbreviations: Data are presented as median (P_25_, P_75_) for nonnormal distributed continuous variables. **p-value*<0.05, ** *p-value*<0.01.

**Supplementary Table S2** MetS Definition

| **Diagnostic criteria** |  | **NCEP ATP III (2001)** | **This study** | |
| --- | --- | --- | --- | --- |
| Central obesity | Male | waist circumference > 102 cm | BMI ≥ 30 kg/m^2^ |  |
|  | Female | waist circumference > 88 cm |  |  |
| Hypertension |  | BP≥ 130/85 mmHg | BP≥130/85 mmHg | or treated with anti-hypertensive drugs |
| Impaired fasting plasma glucose |  | FPG ≥5.6 mmol/L | FPG ≥5.6 mmol/L | or drugs used for treating diabetes |
| Fasting plasma triglycerides |  | TG≥ 150 mg/dL | TG≥ 150 mg/dL | or treated with drugs for the lipid abnormality |
| Low HDL-C | Male | HDL-C <40 mg/dL | HDL-C <40 mg/dL | or treated with drugs for this lipid abnormality |
|  | Female | HDL-C <50 mg/dL | HDL-C <50 mg/dL |  |

Abbreviations: BP, blood pressure; HDL-C, high-density lipoprotein cholesterol; TG, fasting plasma triglycerides; FPG, impaired fasting plasma glucose; BMI, body mass index.

**Supplementary Table S3** The analysis of VOCs-exposed subjects` characteristics

| **Categories** | **Variables** | **non-MetS** | **MetS** | ***P-value*** |
| --- | --- | --- | --- | --- |
| Gender | Male | 609(49.4%) | 657(50.6%) | 0.551 |
|  | Female | 624(50.6%) | 641(49.4%) |  |
| Age |  | 40.78(16.915) | 57.55(15.014) | 0.00** |
| Family monthly poverty level index | | 2.44(1.43) | 2.42(1.37) | 0.101 |
| Race | Mexican American | 164(13.3%) | 161(12.4%) | 0.00** |
|  | Hispanic | 126(10.2%) | 118(9.1%) |  |
|  | Non-Hispanic White | 412(33.4%) | 454(35.0%) |  |
|  | Non-Hispanic Black | 269(21.8%) | 370(28.5%) |  |
|  | Non-Hispanic Asian | 187(15.2%) | 136(10.5%) |  |
|  | Others race | 75(6.1%) | 59(4.5%) |  |
| Education | Less than 9th grade | 56(4.5%) | 122(9.4%) | 0.00** |
|  | 9-11th grade | 135(10.9%) | 147(11.3%) |  |
|  | High school graduate | 263(21.3%) | 335(25.8%) |  |
|  | Some college | 350(28.4%) | 406(31.3%) |  |
|  | College graduate or above | 429(25.8%) | 279(21.5%) |  |
| Smoking | Smoking | 456(37.0%) | 591(45.5%) | 0.00** |
|  | No smoking | 777(63.0%) | 707(54.5%) |  |
| Drinking | Drinking | 1115(90.4%) | 1188(91.5%) | 0.367 |
|  | No drinking | 118(9.6%) | 110(8.5%) |  |
| BMI |  | 26.65(5.834) | 32.77(7.214) | 0.00** |
| BP | Systolic blood pressure | 116.71(15.945) | 132.84(19.993) | 0.00** |
|  | Diastolic blood pressure | 71.66(10.398) | 78.66(12.035) | 0.00** |
| FPG |  | 5.61(1.150) | 6.89(2.452) | 0.00** |
| HDL |  | 55.67(16.024) | 51.14(14.673) | 0.053 |
| TG |  | 93.12(137.363) | 126.50(86.584) | 0.330 |
| Serum VOCs | BDCM | 0.004(0.004,0.004) | 0.004(0.004,0.004) | 0.010* |
|  | Benzene | 0.017(0.017,0.052) | 0.017(0.017,0.041) | 0.007* |
|  | Benzonitrile | 0.106(0.106,0.106) | 0.106(0.106,0.106) | 0.000** |
|  | Cyclohexane | 0.014(0.014,0.014) | 0.014(0.014,0.014) | 0.029* |
|  | Chloroform | 0.009(0.006,0.015) | 0.008(0.006,0.014) | 0.004* |
|  | DBCM | 0.004(0.004,0.004) | 0.004(0.004,0.004) | 0.016* |
|  | Ethylbenzene | 0.017(0.017,0.036) | 0.017(0.017,0.029) | 0.018* |
|  | MIBK | 0.071(0.071,0.071) | 0.071(0.071,0.071) | 0.004** |
|  | 1,1,1-TCA | 0.071(0.071,0.071) | 0.071(0.071,0.071) | 0.024* |
| Urinary VOCs | AMCC | 122(58.2,249.5) | 150.5(76.25,299) | 0.000** |
|  | CEMA | 91.9(46.6,174) | 114(55.875,216) | 0.000** |
|  | CYHA | 1.84(1.84,1.84) | 1.84(1.84,1.84) | 0.038* |
|  | CYMA | 1.7(0.702,25.05) | 1.465(0.655,5.738) | 0.006** |
|  | NADB (DHBMA) | 345(191,546) | 379(231,576) | 0.000** |
|  | 2-HEMA | 0.559(0.559,1.66) | 0.559(0.559,1.25) | 0.000** |
|  | IPM3 cysteine | 3.79(1.64,10.00) | 4.25(1.98,9.615) | 0.031* |
|  | HPMM | 213(118,432) | 240(140,443) | 0.007** |
|  | TTCA | 7.90(7.90,18.50) | 7.90(7.90,22.8) | 0.001** |
|  | t,t-MA | 46.4(16.45,113) | 48.55(21.675,114) | 0.027* |

**Abbreviations:** BMI: body mass index; BP, blood pressure; HDL-C, high-density lipoprotein cholesterol; TG, fasting plasma triglycerides; FPG, impaired fasting plasma glucose; Data are presented as median (P_25_, P_75_) for nonnormal distributed continuous variables, or count (%) for categorical variables. * *p-value*<0.05, ** *p-value*<0.01.

**Supplementary Table S4** The analysis of subjects` behavioral risk factor

| **Variable** | **Category** | **non-MetS** | **MetS** | **X^2^** | ***P-value*** |
| --- | --- | --- | --- | --- | --- |
| Attached garage | Yes | 481 | 511 | 4.457 | 0.108 |
|  | No | 733 | 750 |  |  |
|  | Unclear | 18 | 34 |  |  |
| Store paints or fuels inside home | Yes | 365 | 381 | 3.886 | 0.143 |
|  | No | 844 | 874 |  |  |
|  | Unclear | 23 | 40 |  |  |
| Use moth balls or toilet deodorizers | Yes | 219 | 308 | 18.042 | 0.00** |
|  | No | 986 | 944 |  |  |
|  | Unclear | 27 | 43 |  |  |
| Inhale smoke for 10 min in the past 3 days | Yes | 263 | 247 | 6.149 | 0.046* |
|  | No | 950 | 1012 |  |  |
|  | Unclear | 19 | 36 |  |  |
| When did you last inhale smoke for 10 min | Today | 118 | 130 | 5.570 | 0.134 |
|  | Yesterday | 96 | 74 |  |  |
|  | More than 2 days | 49 | 42 |  |  |
|  | Unclear | 969 | 1049 |  |  |
| Cook with natural gas | Yes | 270 | 286 | 4.919 | 0.085 |
|  | No | 942 | 971 |  |  |
|  | Unclear | 20 | 38 |  |  |
| Pump gas into your car | Yes | 373 | 366 | 3.809 | 0.149 |
|  | No | 838 | 894 |  |  |
|  | Unclear | 21 | 35 |  |  |
| Spend time in pool, hot tub, steam room | Yes | 34 | 24 | 6.909 | 0.032* |
|  | No | 1180 | 1236 |  |  |
|  | Unclear | 18 | 35 |  |  |
| Use dry cleaning solvent in the last 48 hours | Yes | 40 | 34 | 5.499 | 0.064 |
|  | No | 1174 | 1226 |  |  |
|  | Unclear | 18 | 25 |  |  |

**P-value*<0.05, ** *P-value*<0.01.

**Supplementary Table S5** The Sensitivity analysis in linear regression

| **Factor** | **AME** | **SE** | **z** | ***P-value*** | **lower** | **upper** |
| --- | --- | --- | --- | --- | --- | --- |
| AMCC | 0.0332 | 0.0146 | 2.2787 | 0.0227* | 0.0046 | 0.0618 |
| CEMA | 0.0802 | 0.0156 | 5.1332 | 0.0000** | 0.0496 | 0.1109 |
| CYHA.cysteine | -0.0035 | 0.0166 | -0.2133 | 0.8311 | -0.036 | 0.029 |
| CYMA | -0.0296 | 0.0094 | -3.137 | 0.0017* | -0.0482 | -0.0111 |
| HPMM | -0.006 | 0.0236 | -0.2525 | 0.8007 | -0.0523 | 0.0403 |
| IPM3.cysteine | 0.0092 | 0.0161 | 0.5721 | 0.5673 | -0.0224 | 0.0408 |
| NADB | -0.03 | 0.0241 | -1.2456 | 0.2129 | -0.0772 | 0.0172 |
| t.t.MA | 0.0095 | 0.0081 | 1.1647 | 0.2441 | -0.0065 | 0.0254 |
| TTCA | 0.0039 | 0.0097 | 0.3971 | 0.6913 | -0.0152 | 0.0229 |
| 2-HEMA | -0.0262 | 0.0149 | -1.7643 | 0.0777 | -0.0553 | 0.0029 |

**P-value*<0.05, ** *P-value*<0.01.
